# Hematological Inflammatory Markers Across Neurodevelopmental Disorders: Preliminary Findings of an Observational Retrospective Study

**DOI:** 10.3390/brainsci15090937

**Published:** 2025-08-28

**Authors:** Raffaele Garotti, Maria Pia Riccio, Chiara Staffa, Mariangela Pezone, Carmela Bravaccio

**Affiliations:** 1 Department of Mental and Physical Health and Preventive Medicine, University of Campania “Luigi Vanvitelli”, 80131 Naples, Italy; garottiraffaele@gmail.com; 2 Department of Maternal and Child Health, Child Neuropsichiatry, AOU Federico II, 80131 Naples, Italy; 3 School of Medicine, Federico II University, 80131 Naples, Italy; chiara.staffa@gmail.com; 4 Department of Medical and Translational Sciences, Child Neuropsychiatry, Federico II University, 80131 Naples, Italy; m.pezone01@gmail.com (M.P.); carmela.bravaccio@unina.it (C.B.)

**Keywords:** neurodevelopmental disorders, systemic inflammation, blood cell count, hematological ratios

## Abstract

**Background/Objectives:** Alterations in immunoinflammatory activation may constitute a pathogenetic mechanism in neurodevelopmental disorders (NDDs). Blood cell count (CBC) parameters and hematological inflammatory indices (neutrophil-to-lymphocyte ratio, lymphocyte-to-monocyte ratio, platelet-to-lymphocyte ratio) are now assuming a greater role as potential biomarkers for NDDs. **Methods:** In this retrospective observational study, we gathered data on 135 medication-free individuals aged 6 to 17 years: 90 with NDDs (34 with autism spectrum disorder (ASD), 29 with attention-deficit/hyperactivity disorder, 14 with intellectual disability, and 13 with tic disorder) and 45 typically developed controls. The variables analyzed were compared using analysis of variance including Bonferroni posthoc testing for pairwise comparisons Significance was defined as *p* < 0.05. **Results:** The analysis of variance revealed statistical significance for all evaluated CBC parameters, as well as for the lymphocyte-to-monocyte ratio. Notably, subjects with ASD exhibited increased values of neutrophils, lymphocytes, monocytes, and eosinophils compared to both typically developing subjects and other NDDs. The lymphocyte-to-monocyte ratio was found to be lower in the tic disorder group compared to typically developing subjects. The elevated lymphocyte and monocyte levels in ASD subjects might reflect chronic low-grade inflammation. **Conclusions:** Consistent with the evidence in literature, statistically significant differences between the NDD group and typically developed subjects in the CBC parameters were found. The principal limitations of this investigation are the restricted sample size and the exclusion of specific NDD subtypes. Future research is needed to evaluate CBC parameters and inflammatory indices in a broader spectrum of NDDs to better understand the immunoinflammatory response specific to each disorder.

## 1. Introduction

Neurodevelopmental disorders (NDDs) include heterogeneous conditions characterized by alterations in one or more areas of child development (e.g., social, cognitive, language, and others). These disorders typically emerge during the early stages of development and are associated with impairments in personal, social, school, or work functioning [1]. Despite their nosographic classification, these disorders frequently exhibit overlapping symptomatology, which may hinder the establishment of a clear-cut diagnosis [1]. According to the *Diagnostic and Statistical Manual of Mental Disorders*, Fifth Edition, Text Revision (DSM-5-TR), NDDs are classified into: autism spectrum disorder (ASD),attention-deficit/hyperactivity disorder (ADHD),intellectual disability (ID), communication disorders, specific learning disorders (SLDs) and movement disorders (including tic disorder (TD)) [2]. Recent evidence suggests that neuroinflammation may play a crucial role as a pathogenetic driver in neurodevelopmental disorders (NDDs). Specifically, preclinical studies have demonstrated that dysregulated levels of pro-inflammatory cytokines, such as interleukin 6 (IL-6) and tumor necrosis factor alpha (TNF-α), can induce persistent alterations in neural development [3]. It is also well established that microglia (resident in the central nervous system) can respond to various insults: it starts and subsequently maintains the inflammatory process. Microglia thereby represents one of the cell types most significantly involved in neural inflammation [4]. The question of whether neuroinflammation originates locally within the central nervous system or arises as a consequence of systemic inflammation remains a subject of ongoing debate. Nonetheless, systemic inflammation has been investigated in neurodevelopmental senses as a potential pathogenetic factor. Specifically, it is understood that cytokines and interleukins can cross the blood–brain barrier and that this process is amplified by inflammation itself. In addition to active and passive transport mechanisms, inflammation increases the permeability of the blood–brain barrier, facilitating an increased influx of pro-inflammatory molecules [5]. Despite this, it is well known how systemic inflammation assumes a “transdiagnostic” role and enhances the likelihood of developing psychiatric disorders and NDDs through the dysregulation of certain brain circuits, including the ones responsible for motor control, in response to external stimuli and for gratification [6]. Recently, blood cell count has assumed a prominent role as a marker of inflammation in NDDs. Numerous variables may serve as potential markers of systemic inflammation, such as the neutrophil-to-lymphocyte ratio (NLR), the lymphocyte-to-monocyte ratio (LMR) and the platelet-to-lymphocyte ratio (PLR). Various authors have examined possible differences in hematological markers of inflammation in NDDs. Notably, a meta-analysis by Arteaga-Henríquez, G. et al. encompassing 25 studies revealed that subjects with ASD show a significantly higher white blood cell (WBC) count and higher neutrophil (N) and monocyte (M) concentrations compared to healthy controls [7]. Likewise, Gędek, A. et al. observed elevated NLR and PLR values in subjects with ADHD compared to healthy controls through a meta-analysis of five studies,. Their analysis showed no differences in LMR values and no significant variations across specific ADHD subtypes [8]. Within NDDs, ASD and ADHD stand out as the most extensively researched disorders with the strongest current evidence base. Nevertheless, recent evidence suggests an increase in PLR and NLR even in individuals with SLD when compared to healthy controls [9]. Recently, Kara, M. Z. and Kul M. also evaluated complete blood cell count (CBC) differences between individuals with TD and healthy controls. Among their findings, there was a significant increase in platelet (PLT) count in the TD group compared to the healthy controls [10]. The present study aimed to evaluate differences in CBC and related inflammatory indices in a cohort of subjects with NDDs when compared to healthy subjects. We hypothesized that NDD subjects show alterations in CBC as well as hematological inflammatory indices when compared to typically developing subjects (TDSs). One of the main aims was also to assess whether individual NDDs may present characteristic alterations in the hematological profile that could represent a biological marker of specific clinical conditions. Building upon the current scientific literature, our goal was to broaden the scope of comparison for hematological parameters, specifically by also evaluating individual NDDs against each other.

## 2. Materials and Methods

### 2.1. Study Population

The present study used a cross-sectional design and involved retrospective data collection. Participants were selected from those who attended the Interdepartmental Program of Child Neuropsychiatry at the Federico II Hospital in Naples, Italy, from January 2020 to September 2024. The following inclusion criteria were used to select the subjects: diagnosis of NDD made according to DSM-5 and DSM-5-TR criteria, age between 6 and 18 years, and completion of hematochemical and psychodiagnostic investigations in our ward. The following exclusion criteria were applied: no diagnosis of NDD according to DSM-5 and/or DSM-5-TR, age below 6 years or above 18 years, presence of psychiatric comorbidities or co-occurring diagnoses of two NDDs, history of previous infection in the two weeks preceding the blood tests and/or an ongoing acute infectious state, diagnosis of systemic inflammatory and/or immune diseases (e.g., juvenile rheumatoid arthritis, Crohn’s disease, ulcerative colitis),diagnosis of concomitant neurological diseases (e.g., epilepsy), known genetic syndrome, ongoing psychopharmacological treatment, and failure to undergo blood tests and/or psychodiagnostic evaluation at our facility. For all subjects, a psychodiagnostic evaluation using standardized tests was conducted to confirm the diagnostic hypothesis. For typically developing subjects (TDSs), the same inclusion and exclusion criteria were used, with the exception of the presence of an NDD diagnosis.

The final sample consisted of 135 subjects after screening 350 subjects. The study sample was composed of 34 ASD subjects (25.2%), 29 ADHD subjects (21.5%), 14 ID subjects (10.4%), 13 TD subjects (9.6%), and 45 TDSs (33.3%).

### 2.2. Study Procedure

For all subjects selected for the study, the parameters obtainable from the CBC were assessed. These parameters were analyzed after the collection of peripheral venous blood samples, which had already been performed for clinical evaluation of the subjects. Specifically, EDTA-enriched test tubes were used for CBC analysis. The variables analyzed were WBC, total red blood cell (RBC), PLT, N, lymphocyte (L), M, eosinophil (E), and basophil (B) counts. In addition, the inflammatory indices derived from the CBC parameters were also analyzed, specifically the NLR, PLR and LMR. We considered only analyses performed at our facility’s laboratory in order to reduce potential bias linked to the study procedures.

### 2.3. Statistical Analysis

For all variables under study, the normality of the distribution was assessed by means of Shapiro–Wilk tests. All study variables were found to be non-normally distributed following preliminary assessment. A preliminary descriptive analysis of the sample was performed (means and standard deviation for normally distributed parametric values, medians for non-normally distributed parametric values, and frequency for non-parametric values). Prior to conducting the analysis of variance, the homogeneity of variances was assessed using Levene’s test. Variance homogeneity was observed only for PLT, E, NLR, and PLR parameters (*p* < 0.05). Despite this violation of the homoscedasticity assumption, we nonetheless opted to perform a one-way ANOVA. This decision was taken because comparing the means of the parameters was considered among the most relevant information for the study’s objectives. To enhance the robustness of the analysis and mitigate the impact of heteroscedasticity and non-normal distribution, a stratified bootstrap procedure by sex and age was implemented [11]. This procedure generated 5000 resamples. For statistical inference, bias-corrected and accelerated (BCa) confidence intervals were calculated. Specifically, the comparison between a pair of groups was considered statistically significant if their BCa confidence intervals did not contain zero. Bonferroni’s test was set as the posthoc correction for multiple comparisons among individual groups. Furthermore, analyses were performed by applying a frequency weight based on the subject’s age: this aspect allowed us to increase the statistical contribution of older subjects compared to younger ones. This weighting was performed given the progressive normalization of CBC parameters with increasing age [12].

In the results section, only the statistically significant pairs are presented. Overall, results with a *p*-value < 0.05 were considered statistically significant. Analyses were conducted using the Statistical Package for the Social Sciences Software, version 29.0.

### 2.4. Ethical Considerations

The study was conducted in accordance with the Declaration of Helsinki (2013). A favorable opinion was obtained from an independent ethics committee before the study was carried out. For all subjects, informed consent was signed by the parents/caregivers before the start of the diagnostic assessments at our facility. This informed consent also provided availability for the anonymous use of the subjects’ data for research purposes.

## 3. Results

The descriptive analyses relating to age and sex are summarized in Table 1. A one-way analysis of variance comparing the study groups with respect to age was conducted, which demonstrated that age was different among groups in our sample (F 4.14, *p* < 0.004). Furthermore, to evaluate whether there was a homogeneous distribution of subjects based on sex, a chi-squared analysis was performed, which revealed a non-homogeneous distribution (chi-squared 20.66, *p* < 0.01). The CBC values and inflammatory indices were then characterized considering the diagnosis. The values are summarized in Table 2. As specified in the Statistical Analysis section, one-way ANOVA tests were performed to evaluate potential differences in the distribution of the parameters under study. The results of the analysis of variance for the CBC parameters are listed in Table 3. A statistically significant difference was observed for all evaluated CBC parameters. The results of the pairwise comparisons between individual groups for the CBC parameters are summarized in Table 4. The ASD group exhibited a higher number of granulocytes (both N and especially E) compared to both the TDSs and other NDDs (such as ADHD and ID for N). Furthermore, an increase in L in ID subjects compared to TDSs was also notable. Table 5 lists the results obtained from the one-way ANOVA for the hematological inflammatory markers. Statistical significance in the analysis of variance was found only for the LMR index (results are summarized in Table 5). From analysis of the results of the comparison between individual groups (summarized in Table 6), significance relative to TDSs was observed only for the TD group (which showed a lower value). Conversely, this parameter was increased in the ASD group compared to the ADHD and TD groups.

## 4. Discussion

### 4.1. CBC Values

The present study focused on evaluating potential differences in CBC and inflammatory indices in subjects with NDDs. In our sample, we found different WBC, RBC, PLT, N, L, N, E, and B concentrations after dividing the sample into the different study groups. Our observation regarding the WBC parameter aligns with findings in the literature: a meta-analysis by Arteaga-Henríquez G. et al. indicated generally higher WBC counts in ASD subjects compared to TDSs [7]. This parameter was also elevated in the ASD group compared to the other NDD groups. However, results concerning the comparison between ASD and ADHD contrast with the literature, since previous evidence reported no significant differences in WBC, L, or B parameters in a direct comparison of an adolescent clinical sample [13]. Moreover, the increase in L count contradicts existing literature that found lower L concentrations in ASD compared to controls [14] or no difference [15]. Despite these prior findings, this observation could align with the pathophysiological model of chronic inflammation: chronic inflammatory processes involve continuous recruitment of mononuclear cells (L and M) to inflamed sites [16]. Thus, the elevated L levels in ASD subjects might reflect chronic low-grade systemic inflammation, a notion further supported by the increased M levels observed in the ASD group compared to controls. In particular, during systemic inflammation, monocytes have the potential to infiltrate the central nervous system and subsequently differentiate into macrophages. The inflammatory environment can activate these macrophages, producing more inflammatory mediators and thus sustaining chronic inflammation. This process has been shown to directly affect brain cytoarchitecture (especially within the limbic system) and dopaminergic neurotransmission systems (within the substantia nigra) [17]. Supporting this preclinical evidence, a recent meta-analysis by Ju X.D. et al. investigated the role of monocytes in NDDs. Their analysis revealed increased M levels in the blood of individuals with five different NDDs compared to TDSs, leading to the conclusion that elevated M levels may indicate neurodevelopmental alterations [18]. In relation to this, the finding of increased M levels in ASD subjects within our sample is consistent not only with some clinical studies [19] but also with the meta-analysis conducted by Arteaga-Henríquez G. et al. [7].

Similarly, our findings regarding the quantity of E in our sample diverged from those reported in the literature. Specifically, we observed an elevation in the number of these cells in the blood of individuals with ASD compared to both TD and individuals with TDSs. This observation contrasts with the existing literature, which typically reports no statistically significant differences in the number of E when comparing individuals with ASD and TDSs [15,20]. This finding appears noteworthy, particularly considering the frequent comorbidity observed between ASD and allergic conditions. Specifically, a longitudinal observational study has indicated that children with atopic dermatitis exhibit a higher probability of receiving a subsequent diagnosis of ASD compared to children without this condition [21]. Furthermore, the observed difference between individuals with ASD and TD could imply distinct roles of the immune system not only in the pathogenesis of the disorder but also in the prevalence of pediatric comorbidities. However, there are currently no preceding data in this regard.

Finally, the finding of an increased number of platelets in ASD and ID subjects compared to TDSs could also be significant. Specifically, platelets are actively involved in the regulation and maintenance of inflammation, both through direct action on the vascular endothelium and via the secretion of cytokines and chemokines contained within their granules [22]. Therefore, increased levels in ASD and ID subjects could signal heightened systemic inflammatory activity. Similarly, a close interplay between platelets and N is well known: platelets are in fact involved in activating neutrophils at inflammation sites, leading to the propagation and maintenance of systemic inflammation [23]. This aspect is of particular interest given the finding of increased PLT and increased N in ASD subjects compared to TDSs. Therefore, the rise in various cell lines involved in inflammation and immunity in ASD subjects might suggest complex immunoinflammatory mechanisms involved in the pathogenesis of this disorder. Regarding the criterion of excluding subjects with a history of infection in the two weeks preceding the blood tests, this was set to avoid any alterations in CBC [24].

### 4.2. Hematological Inflammatory Indices

Regarding the hematological inflammatory indices, statistical significance was observed for the LMR index. Compared to TDSs, subjects in the TD group exhibited lower average values. Relative to TDSs, the other groups did not reach statistical significance. However, this parameter was found to be higher on average in ASD subjects compared to ADHD and TD subjects. Moreover, ID subjects presented higher values of this index compared to TD subjects. The finding concerning ASD subjects contradicts the existing literature: one clinical study reported that this value was in fact lower in ASD subjects compared to controls [19]. The LMR also showed an inverse correlation with the intensity of autistic symptoms. At present, there are no studies evaluating this index in ADHD or ID. However, regarding ADHD, some studies have observed that its inverse (monocyte-to-lymphocyte ratio) is increased in this disorder. Therefore, our results might also be discordant with the literature concerning what has been observed for ADHD [8]. While these indices are being investigated for their potential contribution as diagnostic or follow-up biomarkers for certain NDDs, current results remain inconsistent, and the methodologies of different studies are often not directly comparable [7,25].

### 4.3. Strength, Limitations, and Future Perspectives

A notable strength of our study is the multiple comparison of distinct NDDs, an approach relatively rare in current research. Assessing immunoinflammatory changes characteristic of different NDDs could be beneficial not only clinically but also for elucidating patterns of neurobiological changes specific to each disorder. To date, unfortunately, the effort to find diagnostic biomarkers of NDDs has not yielded consistent results. A recent meta-analysis by Cortese S. et al. observed numerous limitations in the various studies that have evaluated possible biomarkers of NDDs, with evidence being fragmented, not always suitable, and often derived from small study samples [26]. The primary limitation of this study arises from the retrospective nature of its data collection. For example, data on the subjects’ pubertal status are unavailable. This is a relevant factor given the significant number of female participants in the sample. The menstrual cycle can affect CBC parameters, causing alterations that could not be controlled due to the retrospective nature of the study. The retrospective nature of the study also led to sample discrepancies regarding sex distribution, with a higher prevalence of male subjects in the NDD group. However, this aspect is partly consistent with the literature: it is known that NDDs, particularly ASD, ADHD, and ID, are more prevalent in males [27]. To address this significant constraint, stringent criteria for both inclusion and exclusion were applied. Consequently, this rigorous methodology led to the ineligibility of a considerable number of potential participants. An additional limitation is the limited availability of individuals with speech disorders and SLD. This constraint restricted the initial aim of including subjects diagnosed with the full range of NDDs. This methodological challenge may impact the overall cross-sectional representativeness of our findings, especially considering the current paucity of data concerning hematological markers in speech disorders. Essentially, the non-inclusion of certain NDDs could affect the generalizability of the observed results. Furthermore, the sample size was limited, particularly when stratified by individual diagnostic categories, which may introduce challenges in generalizing the highlighted results. A final limitation to the present study could be the inclusion of subjects over 6 years of age. This criterion was added to allow better comparison of CBC values between subjects, given the differences in blood counts present under 6 years of age [12]. This criterion poses limitations mainly related to the evidence that possible pathogenetic mechanisms of NDDs are early and may even be established in utero. In recent times, research into immune abnormalities as predisposing factors for neurodevelopmental disorders (NDDs) has increasingly focused on the potential role of maternal immune activation (MIA). MIA, defined as an excessive activation of the maternal immune system associated with a dysregulation of immune and inflammatory mediators, may trigger alterations in synaptic plasticity and an abnormal organization of the central nervous system [28]. One potential hypothesis for study is that MIA might act as a disease primer for psychiatric disorders. Specifically, MIA could increase an individual’s susceptibility to genetic and environmental factors that may contribute to the development of psychiatric disorders like ASD and schizophrenia [29]. Consequently, Ellul P. et al. recently illustrated that, for instance, the NLR parameter is increased in subjects with ASD whose mothers had a history of MIA, in contrast to those whose mothers did not have this risk factor [30]. Concurrently, it has been noted that MIA may directly impact the phenotype of subjects with ASD. More precisely, the presence of MIA could influence the social–relational competencies of individuals with ASD, and this effect seems to be unrelated to the severity of the individual’s autistic symptoms [31]. These aspects are also evident in relation to ADHD: it has been observed that not only is there a strong association between autoimmune diseases and ADHD but also that polymorphisms of inflammation-associated genes are linked to ADHD [32]. Taken together, this evidence therefore confirms how inflammatory mechanisms can impact early childhood neurodevelopment, with involvement even in utero and subsequent effects on systemic inflammatory markers. Considering these findings, an abnormal immunoinflammatory response appears to be an important mechanism in NDDs. Consequently, further research examining the role of hematological inflammatory markers in larger cohorts that include the spectrum of NDDs is warranted. Moreover, investigations into potential variations in immunoinflammatory activation among different NDDs aiming to identify disorder-specific characteristics are also warranted.

## 5. Conclusions

This retrospective study investigated CBC parameters and hematological inflammatory indices in a cohort of individuals with NDDs. An increase in WBCs was observed in individuals with ASD compared to TDSs. Furthermore, elevated L and M levels were also noted, consistent with the hypothesis that ASD pathogenesis may be associated with chronic low-grade inflammation. No significant results were found for other NDD groups when compared to TDSs. The limitations of this study include its retrospective design and the limited sample size. A notable strength, however, is the comparison across various NDDs, an approach not yet prevalent in the existing literature. Given these findings and considering some discrepancies with prior research, further investigations are suggested to define the role of hematological markers in NDDs within the clinical context.

## Figures and Tables

**Table 1:** Age and sex of the subjects in the sample broken down by diagnosis. * Values expressed as means ± standard deviation; age was calculated in years. † Values expressed as n (male/female). The *p*-values associated with the one-way ANOVA of age and the chi-squared test for sex. ASD: autism spectrum disorder; ADHD: attention-deficit/hyperactivity disorder; ID: intellectual disability; TD: tic disorder; TDSs: typically developed subjects.

	ASD	ADHD	ID	TD	TDSs	*p*
Age *	9.1 ± 2.4	9.1 ± 2.9	11.4 ± 3.2	11.8 ± 2.8	10.8 ± 3.1	0.004
**Sex †**	30/4	25/4	9/5	12/1	24/21	<0.001

**Table 2:** Observed CBC values and inflammatory indices for subjects divided according to diagnosis. Values are expressed as medians. ASD: autism spectrum disorder; ADHD: attention-deficit/hyperactivity disorder; ID: intellectual disability; TD: tic disorder; TDSs: typically developed subjects; WBCs: white blood cells; RBCs: red blood cells; PLT: platelets; N: neutrophils; L: lymphocytes; M: monocytes; E: eosinophils; B: basophils. NA: not applicable. The normal range refers to the values considered normal at our laboratory for the pediatric age group (between 0 and 17 years).

	ASD	ADHD	ID	TD	TDSs	Normal Range
**WBCs (/uL)**	8170	6745	7180	6700	6535	4500–11,000
**RBCs (/uL)**	4,890,000	4,930,000	4,930,000	49,00,000	4,830,000	4,000,000–16,000,000
**PLT (/uL)**	296,000	298,000	286,000	267,000	278,000	150,000–450,000
**N (/uL)**	3300	3485	3230	2960	6420	1800–7000
**L (/uL)**	3150	2555	2810	2660	2510	1000–4800
**M (/uL)**	500	470	440	450	425	100–800
**E (/uL)**	320	210	180	160	155	0–450
**B (/uL)**	40	30	40	40	40	0–200
**NLR**	1.1	1.2	1.1	1.1	1.2	NA
**PLR**	94.5	113.2	100.3	122.6	109.5	NA
**LMR**	0.14	0.17	0.15	0.18	0.16	NA

**Table 3:** One-way ANOVA results. * Statistically significant results. WBCs: white blood cells; RBCs: red blood cells; PLT: platelets; N: neutrophils; L: lymphocytes; M: monocytes; E: eosinophils; B: basophils.

	F	*p* Value
WBCs	8.84	<0.01 *
RBCs	8.73	<0.01 *
PLT	2.71	0.031 *
N	4.08	0.03 *
L	6.03	<0.01 *
M	3.62	0.07 *
E	3.42	0.01 *
B	5.57	<0.01 *

**Table 4:** Results of pairwise comparisons between groups for CBC parameters. Only statistically significant pairs are listed. BCa: bias-corrected and accelerated; ASD: autism spectrum disorder; ADHD: attention-deficit/hyperactivity disorder; ID: intellectual disability; TD: tic disorder; TDSs: typically developed subjects; WBCs: white blood cells; RBCs: red blood cells; PLT: platelets; N: neutrophils; L: lymphocytes; M: monocytes; E: eosinophils; B: basophils.

CBC Parameters	Pairwise Comparisons	BCa Interval	
		Lower	Upper
**WBCs**	ASD–ADHD	827.05	2692.77
	ASD–ID	771.31	2644.33
	ASD–TD	1274.87	3200.92
	ASD–TDSs	1040.19	2727.27
**RBCs**	ASD–ID	−582,151.36	−127,455.51
	ASD–TD	−645,170.76	−102,421.57
	ADHD–ID	−514,940.77	−26,413.16
	ADHD–TD	−577,685.08	−12,517.77
	ADHD–TDSs	58,825.19	372,921.01
	ID–TDSs	258,893.87	724,142.37
	TD–TDSs	254,428.99	755,312.03
**PLT**	ASD–TD	5943.22	64,616.79
	ASD–TDSs	10,497.97	65,012.23
	ID–TDSs	4044.88	47,700.64
	TD–ID	−44,933.16	−1169.34
**N**	ASD–ADHD	134.62	1576.68
	ASD–ID	308.30	1833.10
	ASD–TD	565.55	1921.77
	ASD–TDSs	99.96	1503.74
	TD–TDSs	−847.68	−37.94
**L**	ASD–ADHD	255.11	1150.72
	ASD–ID	47.17	860.57
	ASD–TD	286.11	1150.72
	ASD–TDSs	415.32	1180.73
	ID–TDSs	24.67	661.13
**M**	ASD–ID	31.82	154.09
	ASD–TDSs	30.03	140.53
	ID–TD	−107.43	−0.79
**E**	ASD–ADHD	25.52	225.40
	ASD–TD	59.94	277.04
	ASD–TDSs	44.66	236.96
**B**	ASD–ADHD	9.85	34.38
	ASD–ID	7.85	30.65
	ASD–TDSs	5.06	27.02
	ADHD–TD	−20.29	−1.28

**Table 5:** One-way ANOVA results. * Statistically significant. NLR: neutrophil-to-lymphocyte ratio; LMR: lymphocyte-to-monocyte ratio; PLR: platelet-to-lymphocyte ratio.

	F	*p* Value
**NLR**	1.42	0.23
**PLR**	0.82	0.51
**LMR**	3.14	0.015 *

**Table 6:** Results of pairwise comparisons between groups for hematological inflammatory indices. Only statistically significant pairs are listed. BCa: bias-corrected and accelerated; ASD: autism spectrum disorder; ADHD: attention-deficit/hyperactivity disorder; ID: intellectual disability; TD: tic disorder; TDSs: typically developed subjects; LMR: lymphocyte-to-monocyte ratio.

Inflammatory Index	Pairwise Comparisons	BCa Interval	
		Lower	Upper
**LMR**	ASD–ADHD	0.36	2.22
	ASD–TD	0.51	2.52
	ADHD–ID	−2.17	−0.46
	ID–TD	0.63	2.41
	TD–TDSs	−1.58	−0.31

## Data Availability

The datasets analyzed during the current study are available from the corresponding author upon reasonable request.
